# Comprehensive Management of Diabetic Foot Ulcers: Current Evidence and Practice

**DOI:** 10.1111/1753-0407.70221

**Published:** 2026-04-24

**Authors:** Ji Min Kim, Seon Mee Kang, Jung Hwa Jung, Ki Chun Kim, Sanghyun Ahn, Chong Hwa Kim, Tae Sun Park, Ie Byung Park

**Affiliations:** ^1^ Division of Endocrinology and Metabolism, Department of Internal Medicine Chungnam National University Daejeon South Korea; ^2^ Division of Endocrinology and Metabolism, Department of Internal Medicine Soonchunhyang University Cheonan South Korea; ^3^ Department of Internal Medicine Gyeongsang National University Jinju South Korea; ^4^ Institute of Health Science Gyeongsang National University Jinju South Korea; ^5^ Department of Orthopaedic Surgery, Seoul Medical Center Seoul South Korea; ^6^ Division of Vascular Surgery, Department of Surgery Seoul National University Seoul South Korea; ^7^ Division of Endocrinology and Metabolism, Department of Internal Medicine, Sejong General Hospital Bucheon South Korea; ^8^ Division of Endocrinology and Metabolism, Department of Internal Medicine Jeonbuk National University Jeonju South Korea; ^9^ Division of Endocrinology and Metabolism, Department of Internal Medicine Gachon University Gil Medical Center Incheon South Korea

**Keywords:** amputation, diabetic foot, diabetic neuropathy, foot ulcer, peripheral arterial disease

## Abstract

Diabetic foot ulcers (DFUs) represent one of the most severe and resource‐demanding complications of diabetes mellitus, significantly contributing to morbidity through infections, lower‐limb amputations, reduced quality of life, and elevated healthcare expenditures. The etiology of DFUs involves a multifaceted interplay among diabetic neuropathy, peripheral arterial disease, biomechanical abnormalities, and compromised tissue repair mechanisms. Despite advancements in diabetic care, DFUs continue to pose a substantial global health burden characterized by high recurrence rates and suboptimal long‐term outcomes, particularly evident in regions such as China, where recurrence rates within 1 year exceed 30% among previously healed patients. Effective DFU management requires an integrated, multidisciplinary approach beginning with early risk stratification and accurate diagnosis, followed by targeted antimicrobial therapy, meticulous wound management, appropriate mechanical offloading, and comprehensive vascular assessment and intervention. This review synthesizes contemporary evidence and current clinical guidelines concerning DFU epidemiology, diagnostic criteria, and therapeutic strategies, underscoring the critical role of interdisciplinary coordinated care to enhance clinical outcomes and mitigate associated complications.

## Highlights

Multidisciplinary, evidence‐based strategies are critical for the prevention and management of DFUs, which remain a major global health burden. Early identification and comprehensive, timely treatment approaches are essential to improve outcomes and reduce the risk of recurrence and amputation in DFUs.

## Graphical abstract

This figure illustrates a multidisciplinary approach to the management of diabetic foot ulcers, including wound care, metabolic management, infection control, vascular intervention, and surgical treatment. Integration of these components is essential for optimizing healing, preventing complications, and preserving the limb.

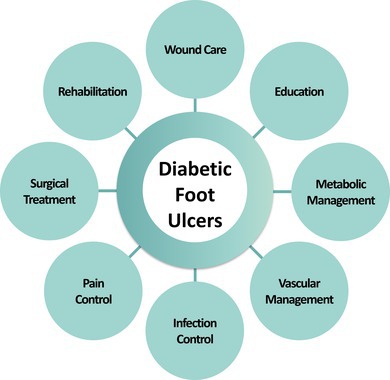

## 1 Introduction

Diabetic foot ulcers (DFUs) are among the most prevalent and severe complications of diabetes mellitus, with a lifetime incidence estimated at approximately 19%–34%. DFUs are strongly associated with increased risks of infection, lower‐extremity amputation, and mortality. The global burden of DFUs continues to rise, driven by the increasing prevalence of diabetes and limitations in early detection and coordinated care.

The pathogenesis of DFUs is multifactorial, typically involving diabetic peripheral neuropathy (DPN), peripheral arterial disease (PAD), and biomechanical abnormalities. These interrelated mechanisms contribute not only to ulcer formation but also to delayed wound healing, a higher risk of infection, and frequent ulcer recurrence.

Despite advances in both preventive and therapeutic strategies, the management of DFUs remains a clinical challenge due to its multifactorial etiology and the need for complex decision‐making and interdisciplinary collaboration. Achieving optimal outcomes requires timely diagnosis, accurate risk stratification, and the coordinated implementation of multidisciplinary interventions including systemic metabolic control, local wound management, and vascular or surgical treatment when necessary.

This review aims to provide an updated overview of epidemiology, pathophysiology, diagnostic approaches, and management strategies. It highlights the essential role of multidisciplinary care in improving outcomes and reducing the clinical and economic burden of diabetic foot complications.

## 2 Epidemiology and Economic Burden of Diabetic Foot Ulcers

Despite increased clinical awareness and guideline‐driven recommendations, DFUs remain a significant complication of diabetes mellitus. The rising global prevalence of diabetes and increased life expectancy have contributed to a growing burden of DFUs, which significantly contributes to patient morbidity and burdens healthcare systems. The lifetime incidence of DFUs is estimated at up to 34% [1]. In China, recent studies report a high burden of DFUs, with an overall incidence ranging from 17% to 43% [2]. High recurrence rates after initial healing pose ongoing challenges for long‐term DFU management, with global pooled data indicating recurrence rates of approximately 40% at 1 year, 60% at 3 years, and up to 65% at 5 years [3]. Approximately 60% of DFUs become infected, significantly increasing the risk of complications, including amputation and mortality [4]. A recent meta‐analysis reported an overall amputation incidence of 22.4% (95% CI, 18.3%–26.5%) among Chinese patients with DFUs [2]. In severe infections, amputation rates may approach 90% [5]. Although global trends indicate a gradual decline in major amputation rates, underserved populations have instead experienced increases of nearly 50% since 2014, particularly among younger Black and Hispanic populations who are disproportionately affected by systemic healthcare disparities [6, 7, 8, 9, 10]. These findings underscore the importance of incorporating social determinants of health and equity‐focused strategies into DFU care.

DFUs place a significant economic burden on healthcare systems and individuals, encompassing costs related to hospitalization, outpatient wound management, surgical interventions, and rehabilitative care. DFU‐related healthcare costs are estimated to be 50%–200% higher than standard diabetes care, reflecting the complexity and intensity of care required. However, such estimates likely underrepresent the full economic impact, as they often exclude losses related to productivity and employment disruptions caused by DFUs [11].

## 3 Etiology, Risk Factors, and Characteristics of Diabetic Foot Ulcers

Diabetic foot disease refers to a broader spectrum of complications beyond DFUs, encompassing DPN, PAD, foot deformities, infections (e.g., cellulitis, osteomyelitis), Charcot neuroarthropathy, and lower‐extremity amputations. DFUs represent the most common and clinically significant manifestation within this spectrum and are defined as full‐thickness wounds below the ankle in individuals with diabetes, regardless of duration.

DFUs result from a multifactorial pathophysiological process, often initiated by repetitive minor trauma to insensitive or preulcerative areas of the foot [12]. DPN impairs protective sensation and proprioception, predisposing individuals to unrecognized injuries caused by abnormal pressure distribution and diminished pain perception. Simultaneously, PAD restricts perfusion, creating a hypoxic tissue environment that further impairs wound healing and increases the risk of ulcer progression [13]. PAD is a contributing factor in approximately 50% of foot ulcers and significantly increases the risk of amputations. These mechanisms frequently coexist, leading to clinical presentations classified as neuropathic (35%–40%), mixed neuroischemic (50%–60%), or purely ischemic (~15%) ulcers [14, 15]. Neuropathic ulcers typically present as well‐defined ulcers with surrounding callus formation and raised edges, commonly located on pressure‐bearing surfaces. In contrast, ischemic lesions often display pale or necrotic wound beds and may present as gangrenous or circular ulcers on distal or friction‐prone sites such as the first metatarsophalangeal joint [13].

In addition to classical mechanisms, recent studies have suggested that persistent hyperglycemia–induced gut microbiome dysbiosis contributes to systemic inflammation and immune dysfunction, thereby playing a role in the pathogenesis of DFUs [16]. Moreover, gut–skin microbiome interactions have been proposed to influence wound healing processes [17].

The development of DFUs is influenced by multiple interrelated clinical risk factors. These include advanced age, male sex, prolonged diabetes duration, poor glycemic control, smoking, and comorbidities. Among these, poor glycemic control is a key modifiable factor, contributing not only to ulcer development but also to delayed healing and progression [18]. In addition, cardiovascular disease has been linked to further impairments in healing and an elevated risk of amputation in patients with DFUs [19, 20].

Chronic kidney disease (CKD) is a well‐established contributor to DFU development and adverse outcomes, with Stage 4 CKD associated with a nearly sevenfold increase in amputation risk [21, 22]. Diabetic retinopathy, particularly when accompanied by visual impairment, further increases the risk of ulceration [23]. Socioeconomic disparities also limit access to preventive services and timely interventions, thereby exacerbating clinical outcomes including higher rates of amputation and mortality [24]. A comprehensive understanding of these etiological factors is essential for targeted prevention and timely clinical intervention.

## 4 Risk Stratification and Clinical Assessment of Diabetic Foot Ulcers

### 4.1 Risk Stratification Principles

Early identification of at‐risk feet or preulcerative lesions is essential for preventing ulceration, reducing amputation risk, and improving outcomes. The American Diabetes Association (ADA) and the International Working Group on the Diabetic Foot (IWGDF) recommend at least annual foot evaluations for all individuals with diabetes, with more frequent assessments for those at higher risk [25, 26]. These evaluations should consider a detailed history of prior foot ulcers or amputations, symptoms suggestive of DPN (e.g., numbness, burning, or tingling) or PAD (e.g., intermittent claudication, rest pain), and additional risk factors including visual impairment, nephropathy, smoking, and poor glycemic control [27].

### 4.2 Comprehensive Clinical Foot Assessment

A comprehensive diabetic foot assessment includes dermatologic, neurologic, vascular, and musculoskeletal components. The initial step involves careful inspection of the feet, focusing on skin integrity, presence of calluses or ulceration, and structural deformities such as claw toe, hammer toe, and Charcot neuroarthropathy. These deformities increase plantar pressure, predisposing to ulcer development [28].

Neurologic assessment should include screening for Loss of Protective Sensation (LOPS), typically using a 10‐g monofilament test. This should be combined with at least one additional method such as vibration perception with a 128‐Hz tuning fork, pinprick sensation, or temperature perception [26]. LOPS, defined as absent monofilament sensation at one or more sites along with another abnormal sensory finding, is strongly predictive of ulceration and amputation [25].

Vascular evaluation includes palpation of the dorsalis pedis and posterior tibial pulses, along with a review of symptoms suggestive of PAD, such as intermittent claudication and rest pain. In cases where PAD is suspected, noninvasive diagnostic tests such as the Ankle‐Brachial Index (ABI), Toe‐Brachial Index (TBI), toe pressure measurements, Doppler waveform analysis, and pulse volume recording should be performed [29, 30]. An ABI value of ≤ 0.9 is indicative of PAD, whereas a value > 1.4 may suggest noncompressible vessels due to arterial calcification. In such cases, toe pressure or TBI provides a more reliable assessment. A TBI < 0.7 or toe systolic pressure < 30 mmHg is generally considered indicative of significant PAD and correlates with poor wound healing outcomes [25].

### 4.3 Classification Systems

A classification system facilitates treatment decisions and improves communication within multidisciplinary teams. Several classification systems have been developed to assess the severity and complexity of DFUs, each with distinct strengths depending on the clinical context.

The Wagner and University of Texas (UT) systems are commonly used in practice. The Wagner system is valued for its simplicity, grading ulcers based on depth and the presence of gangrene, while the UT system provides a more detailed classification by including stages for both infection and ischemia [31]. The SINBAD system, endorsed by the IWGDF, is designed for global clinical use, emphasizing ease of scoring and standardized bedside assessment. The PEDIS system, also developed by the IWGDF, provides a structured format that has been adopted internationally [32]. The WIfI classification, proposed by the Society for Vascular Surgery, stratifies amputation risk and guides revascularization decisions based on wound extent, ischemia, and infection severity [33]. While no single system is universally accepted as the gold standard, each offers distinct advantages depending on the clinical setting, available diagnostic tools, and specific treatment objectives. In clinical applications, these systems also differ in prognostic utility and decision‐making relevance. The SINBAD score is practical in primary care and resource‐limited environments, and has been incorporated into international registries, with higher scores associated with delayed healing in a multicenter prospective cohort study [34]. The WIfI classification is particularly relevant in specialist vascular centers, where it stratifies amputation risk, informs revascularization strategies, and at higher stages provides an indication for referral to vascular specialists. The UT system likewise offers prognostic detail, with advanced grades suggesting the need for surgical or vascular evaluation. The Wagner system, once widely adopted for its simplicity, provides limited prognostic discrimination and is now less frequently used in contemporary clinical practice. By contrast, the PEDIS system is mainly applied in research, being more valuable for standardized documentation and comparability than for direct prognostic guidance. A comparative summary is provided in Table 1.

**TABLE 1:** Comparison of major diabetic foot ulcer classification systems.

Classification system	Parameters	Scoring system	Strengths	Limitations
Wagner	Depth, presence of gangrene	Grades 0–5	Simple, widely used in practice	Limited evaluation of infection, ischemia, and neuropathy, poor predictive value for outcomes
University of Texas (UT)	Depth, infection, ischemia	Grades 0–3 (depth) and Stages A–D (infection/ischemia)	More comprehensive, including infection and ischemia	Slightly more complex, requires thorough examination
SINBAD	Site, ischemia, neuropathy, bacterial infection, area, depth	Each item scored 0 or 1; total score 0–6	Quick bedside tool; globally endorsed, practical in primary care and resource‐limited settings	Limited severity stratification
PEDIS	Perfusion, extent, depth, infection, sensation	Each domain graded 1–4 based on severity	Standardized, facilitates research and consistent documentation	Complex for routine use, limited in low‐resource settings
WIfI	Wound extent, ischemia (based on ABI/TBI), infection	Each domain scored 0 (mild) to 3 (severe); stratified into stages	Comprehensive, enables accurate risk stratification and revascularization planning	Requires vascular assessment, less feasible in primary care

## 5 Management Strategies for Diabetic Foot Ulcers

### 5.1 Conservative and Pharmacological Management for Diabetic Foot Ulcers

#### 5.1.1 Antimicrobial Management of Infected Diabetic Foot Ulcers

Infection is a major contributor to poor outcomes in DFUs, often leading to delayed healing, hospitalization, and amputation. Accurate diagnosis and timely initiation of empiric antimicrobial therapy are essential. For suspected infection, tissue specimens obtained via curettage or biopsy after debridement are preferred for culture and sensitivity testing. Importantly, antibiotics should not be used for clinically uninfected ulcers [35].

Empirical antibiotic therapy should reflect infection severity and the likely pathogens. *Staphylococcus aureus* is the predominant organism in mild, superficial infections, which can typically be managed with oral agents such as dicloxacillin, cephalexin, clindamycin, or amoxicillin/clavulanate. In the absence of clinical improvement, antimicrobial therapy should be re‐evaluated and adjusted based on culture and susceptibility data obtained from deep tissue specimens. Moderate to severe infections, especially those involving deeper structures or associated with systemic signs, are often polymicrobial in nature. These infections warrant the initiation of empiric parenteral broad‐spectrum antibiotics that cover Gram‐positive cocci, Gram‐negative bacilli, and anaerobes. Commonly recommended regimens include vancomycin in combination with agents such as piperacillin/tazobactam, cefepime, ceftazidime, or a carbapenem, with subsequent de‐escalation once pathogens are identified [35]. A detailed summary of empiric antibiotic options according to infection severity and likely pathogens is provided in Table 2.

**TABLE 2:** Empiric antibiotic recommendations for severity of diabetic foot infections.

Severity	Common pathogens	Empiric antibiotic options
Mild^a^	MSSA Streptococcus spp.	Cloxacillin, cephalexin, amoxicillin/clavulanate, ampicillin/sulbactam, fluoroquinolone (alternative option, levofloxacin, or moxifloxacin)
	MRSA (high risk for MRSA)	Trimethoprim‐sulfamethoxazole, doxycycline, clindamycin, linezolid
Moderate to severe^b^	MSSA Streptococcus spp. Enterobacteriaceae	Cefuroxime, cefotaxime, ceftriaxone, amoxicillin/clavulanate, ampicillin/sulbactam
	Anaerobes (if necrosis or limb ischemia)	Amoxicillin/clavulanate, ampicillin/sulbactam, piperacillin/tazobactam, cefuroxime, cefotaxime, or ceftriaxone plus metronidazole or clindamycin
	MRSA (if risk factors present)	Add or substitute vancomycin, linezolid, daptomycin, trimethoprim‐sulfamethoxazole, doxycycline
	*Pseudomonas aeruginosa* (if macerated ulcer or warm climate)	Piperacillin/tazobactam, cefepime, ciprofloxacin, meropenem
	ESBL	Ertapenem, meropenem, imipenem, ciprofloxacin, amikacin, colistin

*Note:* High risk for MRSA: previous MRSA infection or colonization.

MRSA risk factors: prolonged hospitalization, intensive care admission, recent hospitalization, recent antibiotic use, invasive procedures, HIV infection, admission to nursing homes, open wounds, hemodialysis, discharge with long‐term central venous access.

Abbreviations: ESBL: extended‐spectrum ß‐lactamase; MRSA, methicillin‐resistant *Staphylococcus aureus* ; MSSA, methicillin‐sensitive *Staphylococcus aureus* .

^a^ Oral antibiotics for 1–2 weeks are usually sufficient for mild infections.

^b^ Moderate to severe infections typically require 2–4 weeks of therapy, initiated intravenously and followed by oral agents depending on clinical response.

To prevent resistance, both treatment duration and antimicrobial spectrum should be minimized. Antibiotics resolve infections but do not promote wound healing; therapy can be discontinued once clinical signs are resolved. Treatment typically spans 1–2 weeks for mild and 2–4 weeks for severe infections [37, 38]. In cases of diabetic foot osteomyelitis where bone is preserved, a minimum of 6 weeks of targeted antibiotic therapy is recommended. Long‐term suppressive therapy is reserved for cases involving retained hardware or unresolvable necrotic bone. Infectious disease consultation is recommended for complex or refractory infections [35].

#### 5.1.2 Principles of Local Wound Management for Diabetic Foot Ulcers

1. Wound dressing
  Effective wound dressings play a key role in maintaining a moist and protected environment conducive to DFU healing. Current guidelines recommend selecting dressings based on individual wound characteristics, such as exudate level, necrotic tissue, and infection status, rather than favoring any specific type [39]. Hydrogels may modestly improve healing, particularly in wounds with moderate exudate or necrosis [40, 41]. Antimicrobial dressings (e.g., silver‐impregnated) may reduce healing time in selected cases but should not replace systemic antibiotics when infection is present [42].
2. Debridement
  Conservative sharp wound debridement (CSWD) is a cornerstone in managing DFUs, as it facilitates transition from chronic inflammation to active healing. By excising devitalized tissue, it reduces bacterial load, disrupts biofilm, and promotes a granulation‐friendly wound environment [43]. CSWD is typically performed at 48‐h intervals when necrotic tissue or signs of active infection persist [44]. However, recent guidelines recommend that the frequency of debridement should be determined by the clinician based on clinical need, rather than a fixed interval, reflecting the heterogeneity of wound presentations and patient‐specific factors [25]. In cases with overlying calluses, debridement also serves to decrease localized plantar pressure and mitigate risk of ulcer recurrence [45].
3. Nonsurgical offloading strategies
  Mechanical stress, including both shear and direct pressure, plays a pivotal role in the development and persistence of DFUs [46]. Effective offloading is therefore fundamental to ulcer healing [47], particularly in patients with DPN. Total contact casting (TCC) is regarded as the reference standard [39], offering optimal redistribution of plantar pressure and improved healing outcomes compared to removable walking casts and footwear [48]. When TCC is contraindicated or poorly tolerated, alternative modalities such as removable cast walkers, therapeutic footwear, or custom insoles may be considered, particularly in the maintenance phase [49, 50].
4. Adjunctive or emerging therapies
  Several adjunctive or emerging therapies have been investigated as complementary options in the management of DFUs. Negative pressure wound therapy (NPWT) has been associated with higher healing rates and shorter time to healing [51], although the certainty of evidence remains low. According to the IWGDF guideline, NPWT may be considered as an adjunct for postsurgical diabetes‐related foot wounds but is not recommended for non‐surgical ulcers [39]. Recent meta‐analyses further suggest benefits in healing and amputation risk reduction without increased adverse events, suggesting a potential role in selected patients [52]. Among other adjunctive therapies, cellular and acellular matrix products (CAMPs) have demonstrated improved healing outcomes in meta‐analyses [53, 54], although the IWGDF guideline does not recommend their routine use. Recent studies have highlighted advanced hydrogel dressings, designed not only to maintain moisture but also to enable controlled drug delivery and multifunctional activity. Evidence from clinical studies and meta‐analyses suggests that these formulations may enhance healing rates and shorten time to closure compared with conventional dressings, although the overall certainty of evidence remains limited and factors such as cost, handling requirements, and patient‐specific variability continue to constrain their broader clinical application [55, 56]. Platelet‐derived therapies such as platelet‐rich plasma have also been investigated, but results remain inconsistent. An exception is the autologous leucocyte–platelet–fibrin patch, which may be considered when standard care has failed, and appropriate resources are available. Placental‐derived products can also be considered in refractory ulcers, although the supporting evidence remains low. Other cell‐based and growth factor therapies are not recommended in current guidelines due to insufficient evidence [39].

### 5.2 Surgical Management for Diabetic Foot Ulcers

#### 5.2.1 Surgical Intervention for Diabetic Foot Infection

Infections involving deep soft tissue or bone in DFUs frequently require surgical intervention to prevent proximal extension and minimize the risk of limb loss. Prompt surgical evaluation is essential, particularly in the presence of extensive necrosis, fluctuance, abscess formation, or systemic signs of infection [57].

Initial surgical procedures typically involve incision, drainage, and meticulous debridement of devitalized tissue to reduce microbial burden, control local infection, and support wound bed preparation.

In cases of suspected or confirmed osteomyelitis, resection of infected bone may be necessary to achieve adequate source control. Although the optimal extent of bone removal remains debated, accumulating evidence suggests that combining surgical debridement with targeted, culture‐guided antimicrobial therapy improves infection resolution and wound healing outcomes. According to the ADA guideline, timely surgical evaluation is critical in severe infection or ischemic necrosis, and selective bone resection combined with appropriate antimicrobial therapy is recommended for achieving infection control and limb preservation [26]. Surgical decision‐making should be individualized, taking into account the patient's vascular status, overall clinical condition, extent of infection, and functional goals, with the overarching aim of maximizing limb preservation and promoting recovery [57].

#### 5.2.2 Surgical Off‐Loading for Diabetic Foot Ulcers

When structural deformities such as bony prominences or joint malalignment impede ulcer healing or contribute to recurrent plantar pressure, surgical offloading may be warranted [47]. These interventions aim to redistribute mechanical load and promote wound resolution, and are typically reserved for cases where standard offloading methods have proven insufficient. Current guidelines highlight specific techniques, such as Achilles tendon lengthening or flexor tenotomy, which may reduce the risk of ulcer recurrence in patients with diabetes‐related foot deformities [26]. Surgical planning should account for the location and chronicity of the ulcer, biomechanical factors, and overall limb viability, and is ideally conducted in conjunction with vascular assessment to ensure adequate perfusion for postoperative healing [58, 59].

#### 5.2.3 Amputation for Diabetic Foot Ulcers

**FIGURE 1:**
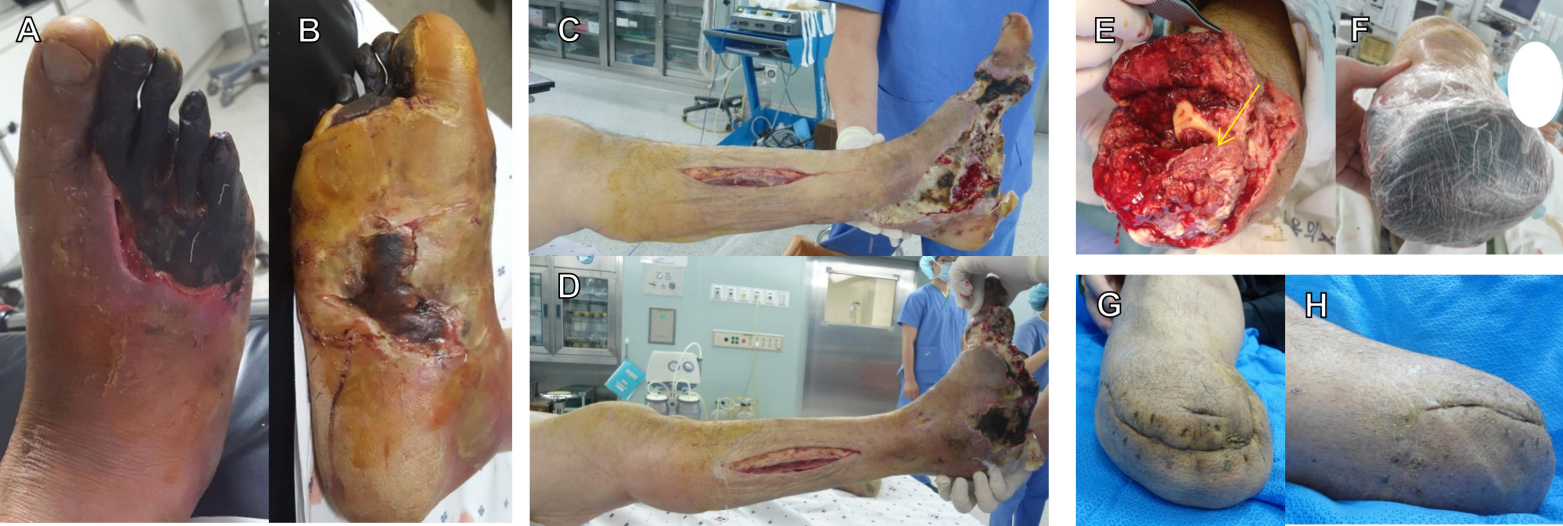
(A) and (B) represent severe necrosis and infection of the foot. In (C) and (D), wide debridement, minor amputation, and fasciotomy areas are observed to control and prevent proximal progression of infection. (E) shows an open amputee end after below knee amputation. Muscle and soft tissues in the posterior compartment are suspected to be infected (arrow). Negative pressure wound therapy (NPWT) was conducted to control infection and salvage the knee joint (F). After repeated debridement and NPWT, the knee joint was successfully salvaged (G, H).

Even with advanced evaluation and management, patients with DFUs complicated by severe infection and/or ischemic necrosis remain at high risk for lower‐extremity amputation [60] (Figure 1). Decision‐making extends beyond wound status to include patient comorbidities, potential mobility, psychological resilience, and support systems [61, 62, 63]. Amputation decisions should prioritize functional recovery and overall quality of life rather than limb preservation alone [26].

### 5.3 Revascularization Strategies for Chronic Limb‐Threatening Ischemia in Diabetic Foot Ulcers

#### 5.3.1 Endovascular Intervention

Revascularization is a critical therapeutic component in the management of DFUs complicated by chronic limb‐threatening ischemia (CLTI) [64]. Timely intervention is associated with improved limb salvage outcomes, while delays significantly increase the risk of major amputation (OR 3.1; 95% CI, 1.4–6.9) [65]. Endovascular treatments for DFUs focus on minimally invasive procedures aimed at improving blood flow and reducing the risk of amputation. Balloon angioplasty—with or without stents, drug‐coated balloons, atherectomy—is less invasive and preferred for frail patients [66, 67, 68, 69]. Recent evidence suggests that drug‐coated balloons are associated with favorable ulcer healing and limb salvage outcomes in patients with CLTI, supporting their potential role in selected cases [70, 71]. However, anatomical challenges (e.g., long‐segment occlusions, heavy calcification) and conflicting evidence on long‐term salvage compared to open bypass remain.

#### 5.3.2 Bypass Surgery and Venous Arterialization

Bypass surgery is a crucial surgical treatment option for DFUs, especially when endovascular interventions are inadequate. When endovascular options are unsuitable, bypass surgery, particularly using the great saphenous vein to tibial or pedal arteries, offers higher limb salvage rates in well‐selected patients [72, 73]. In patients with advanced limb threat (WIfI Stage 3–4), bypass surgery provides superior limb salvage and wound healing outcomes compared with endovascular therapy [74]. In cases of no distal outflow, venous arterialization (surgically directed arterial blood into venous channels) shows promise, with limb salvage rates up to 73% at 2 years in small cohorts [75, 76]. Recent data also indicate that a hybrid approach—combining endovascular popliteal revascularization with above‐knee bypass—achieved a 1‐year primary patency of ~86% and significantly lower major adverse limb events in patients with complex femoropopliteal disease lacking adequate venous conduit [77].

### 5.4 Implications of Invasive Therapeutic Strategies for Diabetic Foot Ulcers in Internal Medicine Practice

**FIGURE 2:**
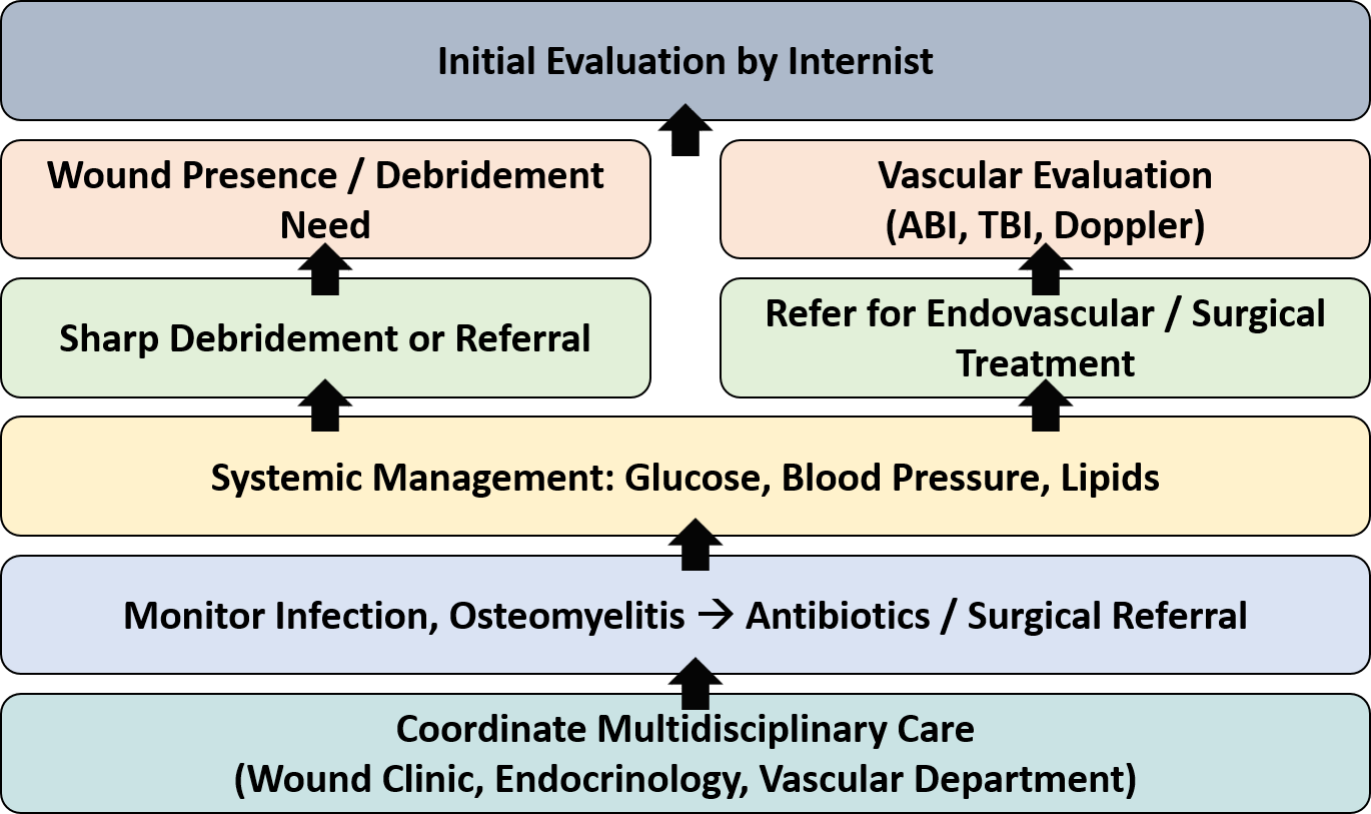
Evaluation and management algorithm of diabetic foot ulcers (DFU). This algorithm provides a structured clinical pathway for internists managing DFUs. It begins with routine screening and risk assessment to detect neuropathy and ischemia. Wound classification guides the intensity of debridement, infection control, and pressure offloading. Vascular evaluation is critical for deciding on endovascular or surgical revascularization. Long‐term care focuses on recurrence prevention through patient education and glycemic optimization. ABI, ankle‐brachial index; TBI, toe‐brachial index.

Internists play a pivotal role in the early identification and coordinated management of DFUs, which are multifactorial in origin (Figure 2). We should recognize the importance of CSWD in converting chronic, nonhealing ulcers into acute healing wounds by removing necrotic tissue and biofilm. Referral to a specialist is recommended when offloading failure is attributable to structural deformities, such as equinus or bony prominences. Vascular assessment using ABI/TBI and Doppler ultrasound is essential to detect CLTI requiring endovascular or surgical intervention. Internists must initiate timely referrals to vascular surgeons to improve limb salvage outcomes and reduce amputation risk [78]. Postrevascularization, internists contribute to long‐term surveillance for restenosis and recurrence. Meticulous systemic control, including glycemic regulation, blood pressure, and lipid management, is essential to promote wound healing and prevent recurrence. Internists should monitor diabetic foot infections and osteomyelitis, facilitating early antibiotic therapy and surgical consultation.

We also coordinate multidisciplinary care with endocrinologists, podiatrists, and wound specialists to optimize clinical outcomes. Although no universal consensus exists regarding which discipline should assume primary responsibility for DFU care, leadership may vary by healthcare system and available expertise. In many centers, internists or endocrinologists coordinate systemic management, while podiatrists, vascular surgeons, and wound specialists take the lead in their respective domains. Effective collaboration is typically achieved through structured multidisciplinary teams, joint clinical pathways, and regular case conferences, which collectively ensure comprehensive and timely patient care. Their integrative approach ensures comprehensive DFU care, improving patient quality of life and reducing healthcare burden.

## 6 Prevention of Diabetic Foot Ulcers

Preventive strategies are essential in reducing the incidence and burden of DFUs. Annual comprehensive foot evaluations are strongly recommended for all patients with diabetes [25, 79], as they enable early detection of peripheral neuropathy, foot deformities, and signs of ischemia [80]. Regular screening facilitates timely intervention and referral to multidisciplinary care teams, which have been associated with a reduced risk of lower‐extremity amputation [81].

Maintaining adequate glycemic control is also crucial. Studies have shown that hemoglobin A1c (HbA1c) levels ≥ 8% significantly increase the risk of amputation [82], while improved glycemic control contributes to better nerve function and lower ulcer incidence [83, 84]. In addition to glucose control, addressing modifiable risk factors such as smoking, hypertension, and dyslipidemia enhances vascular health and reduces the likelihood of ulcer development [85, 86].

Patient education is another key component. While single‐session education may have limited long‐term effectiveness, structured and repeated educational programs have been shown to improve foot care behaviors and may reduce ulcer recurrence, supporting their integration into prevention strategies [25, 87]. A comprehensive prevention strategy should integrate regular clinical assessment, risk factor modification, and patient engagement to minimize the risk of DFUs.

In addition, emerging artificial intelligence–based risk prediction models have shown potential for identifying patients at high risk of ulcer recurrence or amputation, thereby supporting more targeted preventive strategies, although their clinical application remains limited pending further validation [88, 89].

## 7 Conclusion

DFUs constitute a severe complication of diabetes mellitus, significantly impacting patient morbidity, mortality, and healthcare resource utilization worldwide. The escalating global prevalence of diabetes, particularly noticeable in regions such as Korea and China, highlights an urgent need for enhanced strategies encompassing prevention, early detection, and evidence‐based management of DFUs.

Optimal management of DFUs necessitates a comprehensive, interdisciplinary approach integrating expertise from endocrinology, vascular surgery, orthopedics, podiatry, infectious disease, and wound care specialists. Effective treatment relies on systematic risk stratification, meticulous metabolic and vascular assessments, targeted antimicrobial therapy, regular wound debridement, and appropriate offloading techniques. Continuous advancements in therapeutic interventions, including novel dressings, bioengineered tissues, and improved revascularization procedures, are essential to further enhance clinical outcomes and minimize amputation rates.

Preventive strategies grounded in evidence‐based guidelines and tailored patient education initiatives are paramount in reducing DFU incidence and recurrence. Public health efforts should prioritize improving diabetes management practices, addressing modifiable risk factors such as glycemic control, hypertension, dyslipidemia, and smoking cessation, and tackling socio‐economic determinants that limit healthcare access.

In conclusion, addressing the multifaceted challenges associated with DFUs demands an integrated, patient‐centered model characterized by proactive prevention, timely diagnosis, coordinated multidisciplinary care, and rigorous adherence to current clinical guidelines. Implementation of comprehensive care frameworks, supported by ongoing research and robust public health interventions, will substantially mitigate the clinical and economic burden of DFUs, thereby improving patient quality of life and promoting sustainability within healthcare systems globally.

## Author Contributions

**Chong Hwa Kim**, **Tae Sun Park**, **Ie Byung Park:** conceptualization. **Ji Min Kim** and **Chong Hwa Kim:** methodology. **Ji Min Kim**, **Seon Mee Kang**, **Jung Hwa Jung**, **Ki Chun Kim**, **Sanghyun Ahn**, and **Chong Hwa Kim:** writing – original draft. **Tae Sun Park**, **Ie Byung Park:** writing – review and editing.

## Funding

The authors have nothing to report.

## Conflicts of Interest

The authors declare no conflicts of interest.

## Data Availability

Data sharing not applicable to this article as no datasets were generated or analysed during the current study.
